# Treatment of Thrombotic Antiphospholipid Syndrome: The Rationale of Current Management—An Insight into Future Approaches

**DOI:** 10.1155/2015/951424

**Published:** 2015-05-05

**Authors:** Cecilia Beatrice Chighizola, Tania Ubiali, Pier Luigi Meroni

**Affiliations:** ^1^Immunology Research Laboratory, IRCCS Istituto Auxologico Italiano, Via Zucchi 18, 20095 Cusano Milanino, Italy; ^2^Department of Clinical Sciences and Community Health, University of Milan, Via Festa del Perdono 7, 20122 Milan, Italy; ^3^Division of Rheumatology, Istituto Ortopedico Gaetano Pini, Piazza Cardinal Ferrari 1, 20122 Milan, Italy

## Abstract

Vascular thrombosis and pregnancy morbidity represent the clinical manifestations of antiphospholipid syndrome (APS), which is serologically characterized by the persistent positivity of antiphospholipid antibodies (aPL). Antiplatelet and anticoagulant agents currently provide the mainstay of APS treatment. However, the debate is still open: controversies involve the intensity and the duration of anticoagulation and the treatment of stroke and refractory cases. Unfortunately, the literature cannot provide definite answers to these controversial issues as it is flawed by many limitations, mainly due to the recruitment of patients not fulfilling laboratory and clinical criteria for APS. The recommended therapeutic management of different aPL-related clinical manifestations is hereby presented, with a critical appraisal of the evidence supporting such approaches. Cutting edge therapeutic strategies are also discussed, presenting the pioneer reports about the efficacy of novel pharmacological agents in APS. Thanks to a better understanding of aPL pathogenic mechanisms, new therapeutic targets will soon be explored. Much work is still to be done to unravel the most controversial issues about APS management: future studies are warranted to define the optimal management according to aPL risk profile and to assess the impact of a strict control of cardiovascular risk factors on disease control.

## 1. Introduction

Antiphospholipid syndrome (APS) is an autoimmune disorder characterized by vascular thrombosis and/or pregnancy morbidity in the persistent presence of circulating antiphospholipid antibodies (aPL). Antibodies against *β*2-glycoprotein I (anti-*β*2GPI antibodies) and cardiolipin (aCL), together with the functional assay lupus anticoagulant (LA), are the three laboratory tests considered in the revised criteria for the diagnosis of the syndrome. Persistent medium/high positivity, confirmed 12 weeks apart, of at least one of these tests is necessary to diagnose APS [1].

In a large multicentre European cohort of 1000 APS patients, deep vein thrombosis emerged as the most frequent presenting manifestation, and other common vascular events were stroke and pulmonary embolism [2]. The catastrophic variant of APS (CAPS) is a serious aPL-related manifestation, occurring in less than 1% of cases. Multiple small-vessel thrombotic events manifest concomitantly at different anatomic sites in association with a systemic inflammatory response syndrome, which is secondary to the abundant release of cytokines from necrotic tissues [3].

aPL-related vascular events exert a strong clinical impact in terms of morbidity and mortality: it has been estimated that APS affects at least 1% of the general population [2]. In addition, this chronic and disabling condition usually presents in early adulthood: the median age at disease onset is 31 years [4]. Such epidemiological evidence implies that APS diagnosis carries high social and economic costs, making pivotal to correctly manage these patients. The optimal therapeutic approach to APS should aim at attenuating the procoagulant state balancing the side effects of anticoagulation: a careful weighting of risks and benefits should be performed, taking into account the hazard of recurrence as well as of bleeding.

The evaluation of the pros/cons ratio for each treatment option acquires particular importance when evaluating the primary thromboprophylaxis of aPL carriers. In this context, the aPL profile should be carefully considered, taking into account that low-titre and transient aPL positivity does not display a clinical significance, being described even in healthy individuals and in several pathological conditions, such as infections.

On the other hand, anticoagulation is burdened by significant side effects: such poor safety profile of drugs acting on the coagulation cascade explains why novel therapeutic approaches are currently under investigation, in order to identify pharmacological tools specifically counteracting aPL-mediated prothrombotic effects.

## 2. aPL Pathogenic Mechanisms

aPL do not provide merely serum APS biomarkers but rather exert a direct pathogenic role in both vascular and obstetric events. aPL are a heterogeneous family of autoantibodies reacting against proteins with affinity for negatively charged phospholipids (PL). In particular, *β*2-glycoprotein I (*β*2GPI) provides, together with prothrombin, the main epitope targeted by aPL. Three configurations of *β*2GPI have been described: circulating plasma *β*2GPI exists in a circular form; upon binding to suitable anionic surfaces as cardiolipin (CL) and other PL or to LPS, the molecule opens into a J-shaped fish-hook configuration. *β*2GPI consists of 5 domains (D): DI–IV comprise 60 amino acids and contain two disulfide bridges each, while DV is aberrant, as it includes 82 amino acids due to a 6-residue insertion and a 19-residue C-terminal extension cross-linked by an additional disulfide bond. DI has been identified as the most relevant antigenic target involved in *β*2GPI/anti-*β*2GPI antibody binding. This epitope is a cryptic and conformation-dependent structure: in the circular conformation of *β*2GPI, DI interacts with DV and the critical epitope is thus hidden. Several factors might lead to the surface exposition of the critical epitope, such as oxidative stress. Indeed, under oxidative conditions, disulfide bonds form in the molecule leading to the unmasking of the critical B-cell structure [5].

aPL are well accepted to exert a thrombogenic effects* in vitro*; aPL mediate such a thrombophilic state by interfering with both soluble components and cells involved in the coagulation cascade [6]. aPL promote aggregation and activation of platelets, neutralizing *β*2GPI interaction with von Willebrand factor and enhancing the expression of platelet membrane glycoprotein (GP) IIb/IIIa. Moreover, aPL induce a proinflammatory and procoagulant endothelial phenotype upregulating cellular adhesion molecules, promoting the synthesis of endothelial nitric oxide synthase (eNOS) and of proinflammatory cytokines as interleukin (IL)-6 and tumour necrosis factor- (TNF-) *α*. aPL-induced effects on the endothelium are mainly mediated by the reactivity of the autoantibodies with *β*2GPI expressed on the endothelial cell (EC) membrane. Many molecules have been advocated as potential mediators of *β*2GPI interaction with ECs: Annexin A2, Toll-like receptor (TLR) 2 and 4, Heparan-sulfate, and ApoER2'. Lastly, aPL have been shown to significantly increase in both ECs and monocytes the expression of tissue factor (TF), the major initiator of the clotting cascade. There is general agreement that nuclear factor *κ*B (NF*κ*B) and p38 mitogen-activated protein kinase (p38MAPK) are involved in the downstream signalling pathways engaged by aPL in EC and monocyte activation. In addition, aPL have been recently demonstrated to recruit the mammalian target of rapamycin (mTOR) via the phosphatidylinositol 3-kinase- (PI3K-) AKT pathway [7]. However, aPL are not sufficient per se to trigger thrombosis* in vivo*: clotting takes place exclusively in the presence of a second hit (“two-hit hypothesis”) [6].

## 3. The Impact of aPL-Related Vascular Events in the Real Life

### 3.1. Frequency of Thrombotic APS

In a recent work by the APS ACTION, the systematic analysis of 73 relevant papers allowed estimating the aPL positivity rate in the general population with related vascular outcomes as follows: 10% in patients with deep vein thrombosis, 11% in subjects myocardial infarction, and 14% in individuals with stroke [8].

### 3.2. The Strength of Association of aPL with Vascular Events

According to a recent literature revision, the association with aPL appears to be rather solid for vascular events: 54% of studies confirmed the association of aPL with deep venous thrombosis, 55% the association with myocardial infarction, and 71% the association with stroke [9].

## 4. Risk Stratification

aPL carriers do not display all a similar thrombotic hazard; several parameters should be accounted to accurately stratify the risk of developing a vascular event.

### 4.1. aPL Profile

Each aPL profile confers a characteristic thrombotic risk. Among the three criteria aPL test, LA has been appointed as the strongest predictor of clinical events, raising the risk of thrombosis by approximately 4-fold [8, 10].

The thrombotic risk increases with the number of positive aPL tests, with triple positive patients displaying the highest vascular hazard. According to the revised classification criteria, APS patients might be stratified into four categories upon the number of positive aPL tests: category I includes patients with more than one positive test in any combination, while patients with a single positive test should be classified in category II [1]. aPL isotypes should also be considered: IgG are clinically more meaningful compared to IgM [11]. Recently, an increasing interest has been catalysed by IgA isotype, whose role in APS warrants further investigation. Among the novel diagnostic and prognostic tool, the subset of anti-*β*2GPI autoantibodies specifically reacting against DI displays a higher specificity for APS and predicts thrombosis [3].

### 4.2. Associated Autoimmune Conditions

Patients with an underlying systemic autoimmune condition present an excess vascular morbidity, not fully ascribable to traditional cardiovascular risk factors. In particular, aPL positivity has been identified as one of the main determinants of thrombosis among subjects with systemic lupus erythematosus (SLE) [12].

### 4.3. Cardiovascular Risk Factors

The two-hit hypothesis fits well with the clinical observation that thrombotic events occur only occasionally despite the persistent presence of aPL. Consistently, most APS patients experiencing a thrombotic event present concomitant cardiovascular risk factors. In particular, hypertension has emerged as an independent predictor for a first thrombotic event in aPL carriers [11]. Infections have also been found to precede APS onset, and their frequency can be as high as 24% in CAPS patients [13]. Consequently, a careful assessment of cardiovascular status should be accomplished in all aPL-positive individuals: age, diabetes, arterial hypertension, dyslipidemia, obesity, smoking, sedentary lifestyle, hyperhomocysteinemia, Protein C, Protein S, and ATIII deficiency, Factor V Leyden and prothrombin mutations, prolonged immobilization, surgical procedures, and oestrogen use.

### 4.4. Site of Thrombosis

Historically, patients with a first arterial event were regarded at higher risk of experiencing a recurrence, with recurrent event almost invariably involving the same circulatory district [14]. This belief is mainly supported by the only survey analysing arterial and venous events separately, concluding that the risk of recurrence was higher for arterial than venous events [15]. Accordingly, two cohort studies reported a higher incidence of arterial events compared to venous ones: 56 versus 51 recurrences in the EuroAPS cohort (even though venous events were twice more frequent as APS presenting manifestations [2]) and 14 versus 5 in the Singapore cohort [16]. In addition, a case-control study identified previous arterial thrombosis, together with smoking and diabetes, as a predictor of new arterial events [17]. Conversely, in a cohort of high-risk subjects the event at presentation did not predict the site of the recurrence [18].

## 5. Therapeutic Management of Thrombotic APS Manifestations

Some issues in the pharmacological management of thrombotic APS are still subject of a vigorous debate. These criticisms are difficult to solve because of the several limitations flawing the whole literature, including the two randomized studies assessing the efficacy of anticoagulation [19, 20].

Critical items relate to the following issues:

- *Study Design*. Most studies present (i) a retrospective design and (ii) a small sample size.
- *aPL Testing*. Many studies have included patients not fulfilling APS laboratory criteria in terms of (i) number of aPL tests performed; (ii) aPL positivity confirmation; (iii) aPL cut-off.
- *Patients' Selection*. Recruited patients were not stratified upon (i) aPL profile; (ii) cardiovascular risk factors; and (iii) site of thrombosis (arterial versus venous).

To overcome these issues and highlight evidence, two systematic reviews included treatment recommendations for aPL-related thrombotic events [21, 22], while an international Task Force elaborated a consensus document on the primary and secondary thromboprophylaxis in individuals carrying aPL [23].

### 5.1. Pharmacological Agents

The mainstay of the treatment of thrombotic APS is provided by agents that counteract aPL-mediated effects by preventing coagulation.

Antiplatelets as low-dose aspirin (LDASA) are more effective in preventing arterial thrombosis: in the high-flow, high-shear arterial circulation platelet adhesion and aggregation play a major role.

Anticoagulant drugs include vitamin K antagonists (VKA), heparin, and its derivatives.

VKAs act by inhibiting the process of gamma-glutamyl carboxylation of factors II, VII, IX, and X, as well as Protein C and Protein S. The most commonly used VKAs are 4-hydroxycoumarins; among these, warfarin is the most frequently prescribed. VKA treatment presents several pitfalls. These agents have a slow onset of action and a narrow therapeutic window and necessitate frequent INR monitoring. Indeed, they interact with a number of foods and drugs including immunosuppressive agents as azathioprine; VKA activity may fluctuate with alcohol consumption, intercurrent illness, exercise, and smoking. In aPL carriers, VKA monitoring may be rather troublesome because of the variable responsiveness of thromboplastins to LA, even though a multicentre study concluded that LA interference did not significantly affect PT-INR measured with most of commercially available thromboplastins. On the other hand, LA detection in patients on VKA is impaired, thus limiting APS diagnosis among these subjects [24].

Native heparin is a natural anticoagulant whose structure consists of a variable sulfated repeating disaccharide unit, with a molecular weight ranging from 3 to 30 kDa. The inactivation of thrombin by heparin requires at least 18 saccharide units, while the action on factor Xa necessitates only of heparin's pentasaccharide binding site. Interestingly, heparin's activity in APS is not merely attributable to its anticoagulant action. Heparin directly interacts with *β*2GPI: the primary heparin-binding site is located on the second positively charged site within DV of *β*2GPI protein, the domain also deputized to PL binding. Heparin greatly enhanced the plasmin-mediated cleavage of Lys317-Thr318 site in *β*2GPI, resulting in a diminished ability of *β*2GPI to recognize PL and the consequent impairment of the prothrombotic activity of anti-*β*2GPI antibodies [25].

Heparin derivatives as low molecular weight heparin (LMWH) present a lower molecular weight, with the consequent loss of the action on thrombin and a better therapeutic index. At variance to warfarin, these derivatives have no food/alcohol interactions and few drug interactions and do not require routine monitoring thanks to the predictable dose-response relationship. Limitations of LMWH are its subcutaneous administration and side effects as heparin-induced thrombocytopenia (HIT) and osteoporosis.

### 5.2. Bleeding Risk

The risk of bleeding has been shown to progressively increase with the rising of anticoagulation intensity, though this is a nonlinear association. Estimates of the bleeding risk in APS patients are derived from studies evaluating treatment efficacy. A 2007 systematic review evaluating eight studies reported an overall major bleeding rate between 0.57 and 10% per year [22]. However, when considering only recent studies where target INR was 2.0-3.0, the annual bleeding rate dropped down to 0.8–1.6% [26]. Interestingly, the single study specifically addressing the risk of bleeding in APS patients reported no fatal bleeding episodes, and precipitating factors were identified in all cases [27]. It is crucial to note that in APS patients the mortality rate due to thrombosis is higher than the mortality rate due to bleeding. A systematic review considering studies published up to 2007 documented 18 deaths due to recurrent thrombosis, compared with only one decease being attributed to haemorrhage [22]. Most recently, 27 of 1000 patients included in the EuroAPS cohort died during the follow-up period as a consequence of thrombosis, with haemorrhage being the cause of death in only 6 patients [17].

To note, INR fluctuations increase with higher intensity of anticoagulation, contributing to the instauration of not only a hemorrhagic status but also a thrombogenic profile. It is thus crucial to estimate the individual risk of bleeding, in order to prevent complications and improve the quality of life of patients. Independent predictors of major bleeding include INR values above 4.0, concomitant treatment with aspirin, age over 75 years, polypharmacy, history of gastrointestinal tract bleeding, malignancy, lack of education regarding anticoagulation therapy, and leukoaraiosis. In APS patients, uncontrolled blood pressure has also been associated with an increased risk of bleeding. Consequently, special caution should be paid when considering high-intensity anticoagulation or combined aspirin and warfarin therapy.

### 5.3. Venous Thrombotic Events

Starting treatment of venous thrombosis in APS patients follows the recommendations for the management of thrombotic events in the general population: initial therapy consists of UFH or LMWH for at least five days, embraced with anticoagulant agents [28]. With regard to long-term management of patients with venous thrombosis, the optimal intensity and duration of anticoagulation are still matter of debate.

#### 5.3.1. Intensity of Anticoagulation

The management of patients with venous thrombosis envisages two options about anticoagulation intensity: moderate (INR 2.0-3.0) versus high (3.0-4.0) intensity. 

*(i) Moderate-Intensity Anticoagulation*. The effectiveness of moderate-intensity anticoagulation is supported by some lines of evidences.

- Standard anticoagulation at a target INR below 3.0 appeared to confer effective protection against venous recurrences [13, 29–32]. However, it should be noted that many of the above cited studies have recruited patients presenting laboratory tests not fulfilling criteria for full-blown APS.
- The two randomized clinical studies comparing moderate- and high-intensity anticoagulation in patients with a definite APS diagnosis failed to report any difference between the two regimens [19, 33]. Both studies had been specifically designed to demonstrate that high-intensity warfarin offered a better prevention of recurrent thrombosis compared to moderate-intensity anticoagulation. In the study by Crowther on 114 APS patients, the incidence of recurrent thrombosis was even higher among patients receiving high-intensity warfarin (10.7%) compared to those in the moderate-intensity arm (3.4%), although this difference did not achieve statistical significance [19]. Conversely, in the 2005 WASP trial, the recurrence incidences were 11.1% and 5.5% among patients receiving moderate-intensity and high-intensity warfarin respectively [20]. To note, both studies present limited statistical power, because of the inadequate sample size. Another strong bias potentially affected result interpretation: patients in the high-intensity group presented an INR below the target range for over 40% of the follow-up time thus limiting the interpretation of the efficacy of high-intensity regimen. On the other hand, this observation suggests that patients experience more difficulties in keeping INR in the high-intensity range, which may account for the increased frequency of thrombotic recurrences described with the high-intensity regimen in one study. Furthermore, only 18% of patients in the Canadian study and 55% in the WAPS trial had a high-risk aPL profile.
- Not surprisingly, a meta-analysis combining the results of the two randomized studies using Peto's method could not demonstrate any difference in the rate of thrombosis recurrence between the two regimens, although an almost significant excess thrombotic risk was observed with high-intensity anticoagulation [20]. The effectiveness of standard anticoagulation was also supported by a 2007 systematic review by Ruiz-Irastorza considering randomized as well as observational studies [19–22, 29].

*(ii) High-Intensity Anticoagulation*. Some retrospective studies suggested that high-intensity regimen was more effective in preventing thrombotic recurrence compared to either LDASA or low-intensity anticoagulation among unselected APS patients [33, 34] and those with a history of venous thrombosis [35].

More recently, a 45% recurrence rate was observed among triple-positive APS patients with a previous venous events receiving standard anticoagulant therapy over a 6-year period reflecting the poor protection offered by standard-intensity warfarin therapy against recurrent events in this high-risk group [18].

#### 5.3.2. Duration of Anticoagulation

There is currently general consensus to prescribe indefinite anticoagulation to APS patients with a history of venous thrombosis [23]. Nevertheless, an increasing debate has emerged about the potential withdrawal of anticoagulation in a definite subset of aPL patients.

*(i) Indefinite Anticoagulation*. The choice of indefinite anticoagulation for APS patients is supported by the evidence that these patients carry a higher risk of recurrent thrombosis compared to aPL negative subjects, and such thrombotic risk actually increases with time [14]. The persistency of aCL positivity 6 months after a venous event was found to predict thrombotic recurrence and death, with its predictive value increasing with higher titres [32]. In particular, the risk of recurrence was demonstrated to be highest in the six months following discontinuation of anticoagulant drugs [34]. Consistently, cessation of warfarin was shown to induce a thrombotic phenotype because of the recovery of normal levels of procoagulant factors, in turn leading to a stronger interaction between TF and factor VII [36]. 

*(ii) Three-Month Anticoagulation*. In a randomized trial comparing one-month to three-month anticoagulation in patients with venous thrombosis and a transient reversible risk factor, subgroup analysis showed that aPL positivity tested at the time of randomization did not predict venous recurrence [28]. Consistently, over the recent years two-case series demonstrated safe termination of anticoagulation among APS patients who eventually became aPL negative [37, 38]. In this subset of patients, aPL may not play a pathogenic role but rather constitutes an epiphenomenon. It has been therefore proposed that APS patients with a first venous event and a low-risk aPL profile plus a known transient precipitating risk factor could be candidate for 3- to 6-month anticoagulation. In this context, it is advisable to assess ultrasonographically the residual thrombosis and to test D-dimer before suspension of anticoagulation therapy. In the general population, the presence of residual vein thrombosis has been shown to increase the risk of recurrences by 50%, while a negative D-dimer result one month after anticoagulation withdrawal reduces the risk of recurrent thrombosis by twofold. However, the role of ultrasonography and D-dimer test has not yet been assessed in APS population.

### 5.4. Arterial Thrombotic Events

There is overall consensus that APS patients with a previous arterial event deserve indefinite anticoagulation; the optimal intensity of the anticoagulant regimen is still debated.

#### 5.4.1. Moderate-Intensity Anticoagulation

The randomized trials by Crowther and Finazzi recruited also patients with a history of arterial events, even though the latter were underrepresented compared to subjects experiencing venous thrombosis (24% in the study by Crowther and 32% in the trial promoted by Finazzi). As previously pointed out, these two studies were not in support of a superiority of high intensity as compared to moderate-intensity anticoagulation [19, 20]. Consequently, a 2006 systematic review including only these randomized controlled trials recommended moderate anticoagulation [21].

#### 5.4.2. High-Intensity Anticoagulation

The requirement of high-intensity anticoagulation is suggested by the observation that in many studies oral anticoagulation to a standard target INR range of 2.0 to 3.0 was not sufficient in preventing recurrences among patients presenting with arterial events [4, 14, 33, 34, 39, 40].

Consistently, most new thrombotic events occur with an INR below 3.0, as suggested by several cohort studies [11–13, 33, 39, 40] and one randomized controlled study [19, 27, 41].

The choice of a high-intensity regimen is further supported by the higher recurrence risk experienced by APS patients with a history of arterial events, as suggested in some, but not all, studies [2, 15–18]. Indeed, a 47% recurrence rate was observed in a cohort of triple-positive APS patients with a previous arterial thrombosis receiving standard anticoagulation, suggesting its incomplete efficacy [18].

### 5.5. Stroke

Thrombotic events in APS commonly involve the cerebral circulation; stroke is the presenting clinical manifestation in 13% of cases while 7% of APS patients develop a transient ischemic attack at disease presentation. In a large European cohort, stroke accounted for 13% of deaths, at a mean age of 42 years [4]. In case of cerebral arterial thrombosis, the therapeutic scenario also comprises antiplatelet agents.

#### 5.5.1. Low-Dose Aspirin

In the randomized controlled Antiphospholipid Antibodies and Stroke Study (APASS), aspirin at a dose of 325 mg daily was shown to be as effective as low-intensity anticoagulation in the secondary prevention of stroke among aPL-positive patients [42]. However, the APASS study is flawed by some limitations: aPL positivity was not confirmed 12 weeks apart; patients were recruited even when aCL positivity was detected at low-titres. These biases preclude results' extrapolation to patients with definite APS. Indeed, those subjects with baseline positivity for both LA and aCL tended to have a higher event rate (31.7%) than subjects who tested negative for both antibodies (24%).

#### 5.5.2. Low-Dose Aspirin Plus Moderate-Intensity Anticoagulation

In 2009, Okuma first shed light on the combination of LDASA with moderate-intensity anticoagulation as a therapeutic option in stroke patients with a definite diagnosis of APS. In his randomized controlled trial, a lower incidence of recurrent stroke was observed among patients treated with LDASA plus warfarin compared to those receiving LDASA alone, with a cumulative stroke-free survival of 74% versus 25%. However, this study is flawed by important limitations: firstly, the sample size was rather small, no details about aPL profiles of included patients were provided, and limited data on stroke recurrences in each group were available [43].

Given this conflicting picture, an international Task Force could not reach a consensus about the optimal management of arterial thrombosis. Eight out of the 13 members of the Task Force suggested treatment with warfarin with INR over 3.0 for patients with definite APS; the combination of moderate anticoagulation and aspirin was also listed as an option, while LDASA was reserved to stroke patients with a low-risk profile and reversible thrombotic risk factors [23].

Details of the studies addressing therapeutic regimens in thrombotic APS are enlisted in Table 1.

## 6. Additional Therapeutic Tools

Few pharmacological agents have been investigated as alternatives to warfarin; several others have been proposed as adjunctive tools.

### 6.1. Hydroxychloroquine

Hydroxychloroquine (HCQ) is an antimalarial drug with anti-inflammatory and antithrombotic properties. In addition, HCQ has been shown to exert immunomodulatory effects: it prevents activation of TLR3, TLR7, and TLR9, inhibits antigen processing and presentation, and reduces circulating immune complexes [55]. In* in vitro* models of thrombotic APS, HCQ has been demonstrated to inhibit GPIIb/IIIa expression on aPL-activated platelets [56], to reverse the formation of aPL-*β*2GPI-PL bilayer complexes [57] and to prevent the aPL-induced disruption of the Annexin A5 shield [58]. Its antithrombogenic properties have been confirmed in* in vivo* models of APS: HCQ injection in mice induced a dose-dependent decrease in thrombus size [59].

In primary thrombotic APS, HCQ has been evaluated as an adjunctive pharmacological tool: patients receiving a combo regimen comprising HCQ plus oral anticoagulation experienced less recurrences compared to those on anticoagulants only. However, the extrapolation of data is affected by the limitations biasing this work: the study cohort was limited to 40 patients, and the follow-up lasted 36 months only [60]. Nevertheless, HCQ is currently catalysing much attention in APS: an ongoing study is assessing the effect of HCQ on Annexin A5 resistance assay in aPL patients with or without SLE; a randomized controlled trial promoted by the international research organization APS ACTION is evaluating HCQ in the primary prevention of thrombosis in aPL asymptomatic carriers at five-year follow-up [61].

Even though there is limited clinical evidence of its antithrombotic effects in primary APS, treatment guidelines consider HCQ as a potential adjunctive therapy, particularly in consideration of its excellent safety profile [23].

### 6.2. Novel Anticoagulants

Fondaparinux is a synthetic pentasaccharide homologous to heparin binding site; its activity is limited on factor Xa. Fondaparinux has been licensed for thromboprophylaxis, but it has not been yet evaluated in the setting of APS.

Most recently, a novel class of anticoagulants has been synthesized: all are administered orally; these pharmacological agents inhibit a single enzyme of the coagulation cascade, being thus called direct oral anticoagulants (DOA). Dabigatran is a potent, competitive, reversible direct thrombin inhibitor, which binds to thrombin and blocks its interaction with substrates. Direct FXa inhibitors include rivaroxaban, apixaban, and edoxaban. All these agents are highly selective, reversible, competitive, and dose-dependent. They represent an advance over VKA mainly in terms of a better quality of life for patients: since they display a predictable anticoagulant effect, DOA are administered at a fixed dose. In addition, being not metabolized by the cytochrome P450 system, they do not interact with dietary constituents or alcohol and have few reported drug interactions, therefore not requiring routine monitoring of anticoagulant intensity. However, these novel DOA do not allow overcoming some other limitations affecting treatment with VKA. The main issue lies in the significant bleeding risk that any anticoagulant regimen carries, in the absence of an available pharmacological reversal agent [62].

Dabigatran and rivaroxaban have been prescribed to a cohort of 24 French APS patients (11 and 13, resp.); over a median follow-up of 15 months, a single recurrent event was registered [63]. In a UK cohort of 18 APS subjects, rivaroxaban was proved to be safe over 12.9 months [64]. However, caution should be paid when prescribing DOA to APS patients: recently, three cases of thrombotic recurrence upon switching from warfarin to rivaroxaban have been presented [65].

The role of these emerging anticoagulants in APS management is still to be clearly determined: there are few on-going randomized controlled clinical trials evaluating rivaroxaban in the management of APS, as compared to low intensity anticoagulation. The RAPS trial has been promoted by a UK group; it is a phase II/III study that has recruited 156 APS patients with a history of venous thromboembolism. A Spanish phase III trial has been started in Spain on 218 patients with venous or arterial events. Most recently, an Italian trial considering triple positive APS patients only is going to start recruiting [62].

### 6.3. Statins

Statins inhibit cholesterol synthesis in the mevalonate pathway by blocking the 3-hydroxy-3-methyl-glutaryl-coenzyme A (HMG-CoA) reductase. The use of statins in the treatment of APS might thus be beneficial in the prevention of thrombosis since hypertriglyceridemia and low HDL cholesterol levels provide the most frequent cardiovascular risk factor reported in APS patients [66]. However, these pharmacological compounds have also been shown to exert a wide array of additional pleiotropic antithrombotic and anti-inflammatory effects in APS.* In vitro*, fluvastatin, simvastatin, and rosuvastatin have been demonstrated to inhibit TF synthesis in EC [67]; fluvastatin and simvastatin were both reported to suppress anti-*β*2GPI antibody-induced endothelial adhesiveness and to reduce monocyte adhesion to the endothelium [68] while rosuvastatin inhibited the upregulation of VCAM induced by aPL [69].* In vivo*, fluvastatin reduced the size of the thrombus induced by aPL infusion and the leukocyte adhesion to EC. These findings have been later confirmed in* ex vivo* studies. A trial in 42 patients with APS treated with fluvastatin for 30 days reported a decline in several thrombogenic and inflammatory mediators in monocytes [70]. More recently, Erkan observed a significant reduction of half of the evaluated proinflammatory and procoagulant parameters (IL1*β*, VEGF, TNF*α*, IP10, CD40L, and TF) in a cohort of 41 aPL asymptomatic carriers after three months of treatment with fluvastatin [71].

### 6.4. Vitamin D

*In vitro*, vitamin D exerts an antithrombotic and immunomodulator function by inhibiting anti-*β*2GPI antibody-mediated TF expression [72]. Retrospective studies indicated that the prevalence of vitamin D deficiency in APS patients ranges from 10 to 50%, while insufficiency may occur in up to 70% of patients [8, 72–75]. Low vitamin D levels correlate with arterial and venous thrombosis as well as with noncriteria APS manifestations [72, 73, 76]. Conversely, most studies supported no association between low vitamin D levels and obstetric APS [72–74, 76].

### 6.5. Sirolimus

Sirolimus, an mTOR inhibitor, has been used in patients with APS nephropathy undergoing kidney transplantation. Patients receiving sirolimus in the posttransplant immunosuppressant regimen developed no vascular lesion recurrence. Compared to those in the standard regimen arm, patients on sirolimus had a higher rate of functioning allograft at 144 months (70% versus 11%) and a decreased vascular proliferation on biopsy [7].

## 7. Refractory Thrombotic APS

A significant rate of APS patients develops recurrent events despite adequate treatment. In past prospective studies, the yearly incidence of recurrent events among APS subjects receiving antithrombotic therapy ranged between 3 and 24% [29, 30, 58], with this rate even increasing up to 52–69% in retrospective studies [31, 32]. Recurrences yield an important mortality; unfortunately, univocal recommendations to manage these situations are still warranted. Firstly, it should be assessed whether the thrombotic event occurred at a subtherapeutic INR range. If the INR is found to be lower than the target, it can be considered to continue with a moderate-intensity regimen, keeping the INR in the therapeutic range.

Additional therapeutic strategies include the following.

### 7.1. High-Intensity Anticoagulation

If the INR was within the therapeutic range at the time of the recurrence, one option consists in increasing the intensity of anticoagulation. Notably, there is no current evidence in support of this strategy, as both randomized clinical studies by Crowther and Finazzi excluded patients who had a thrombosis while taking warfarin, thus preventing any conclusion about the efficacy of high-intensity anticoagulation in this setting [19, 20].

### 7.2. Low-Dose Aspirin

In patients with arterial events, a potential option is provided by the addition of LDASA to anticoagulant treatment. However, it should be considered that this combination is burdened by a higher bleeding risk. Moreover, very limited evidence is available: only a small, low-quality randomized controlled trial has shown that combination therapy was more effective than aspirin alone in the secondary prevention of aPL-related stroke [43].

### 7.3. Dual Antiplatelet Treatment

Dual antiplatelet treatment (different combination of LDASA, ticlopidine, clopidogrel, and cilostazol) has been recently proposed: a Japanese study on 82 APS patients with refractory arterial events documented no recurrences among those subjects receiving dual antiplatelet agents [44].

### 7.4. Low Molecular Weight Heparin

According to the evidence-based consensus guideline formulated at the 13th International Congress on Antiphospholipid Antibodies, long-term LMWH may also be considered as a safe and effective alternative to warfarin. Details of the reports in support of this therapeutic option in APS are listed in Table 2 [45–48, 77]. Consistently, the 2003 CLOT study stated that dalteparin was even more effective than warfarin in reducing the risk of recurrent embolic events among cancer patients [77].

### 7.5. Intravenous Immunoglobulins

*In vivo* and* in vitro* models suggest the therapeutic potential of intravenous immunoglobulins (IVIg) in APS. IVIg were shown to inhibit aPL, by partially neutralizing LA activity and preventing aCL binding to CL by Fab. IVIg exert an anti-idiotype activity, with inactivation of idiotype-bearing B-cell clones. Furthermore, IVIg have been demonstrated to increase IgG catabolism, to modulate complement activation, to block Fc*γ* receptor on macrophages, and to downregulate proinflammatory cytokines [78]. Treatment with IVIg resulted in an inhibition of aPL thrombogenic effects, with a reduction of circulating aCL levels [79]. There are few reports about successful treatment with IVIg in the management of aPL-related clinical manifestations, mainly haematological (thrombocytopenia, haemolytic anaemia, and hypoprothrombinemia). Treatment response was observed in all cases with a single exception [80]. Recently, IVIg were found to be effective in preventing thrombosis in 7 patients in addition to conventional therapy [81], as well as in a cohort of 5 patients with refractory APS in a five-year open study [82].

### 7.6. Anti-B-Cell Agents

The pivotal role exerted by B cells in APS has been progressively deciphered: B lymphocytes contribute to APS etiopathogenesis by producing autoantibodies, inducing the formation of germinal centres and the synthesis of cytokines. Accordingly, in NZW x BXSB mice, treatment with IgG against B-cell activating factor (BAFF) receptor did not prevent the development of aCL even though it prevented aPL-related thrombotic vasculopathy, prolonging survival [83]. In the same murine models, IgG against cytotoxic T-lymphocyte antigen 4 immunoglobulin (CTLA4) affected initiation but not development of APS. These data suggest the potential efficacy of belimumab and abatacept in APS [84]. Interestingly, in belimumab-treated patients with SLE a positive-to-negative conversion rate was reported for aCL [85]. To date, clinical experience of B-cell inhibitory agents in APS patients is restricted to the use of rituximab, a chimeric monoclonal antibody targeting CD20 on the surface of B cells. Successful treatment with rituximab has been reported in anecdotal reports and in one case series from the BIOGEAS registry. In this multicentre Spanish registry, a therapeutic response was observed in 92% of 12 cases [86]. In 2012, a review collected all the published cases, identifying 27 APS patients treated with rituximab [87]. The anti-CD20 monoclonal resulted in a decrease of aPL titres; among those receiving rituximab because of thrombotic recurrences, clinical improvement was observed in all cases. Moreover, rituximab was beneficial for a plethora of aPL-related clinical manifestations. In this regard, an open-label phase IIa descriptive pilot study (RITAPS) has been carried out in 20 patients with noncriteria APS manifestations refractory to conventional treatments [88]. Rituximab resulted to be effective in controlling some but not all noncriteria manifestations, without substantial change in aPL profile. Notably, caution should be paid to its use in APS: episodes of severe acute thrombotic exacerbations (lacunar infarctions and transverse myelitis) have been reported in two APS/SLE patients receiving rituximab [89].

## 8. Catastrophic APS

Although CAPS is a rare event, the high mortality rate makes this a clinically relevant issue [4]. Most of the available evidence comes from the CAPS registry, a web-based international registry of CAPS patients created by the European Forum on anti-phospholipid antibodies.

### 8.1. Combination Therapy

In CAPS registry, the most effective therapeutic approach comprised anticoagulation, corticosteroids, and plasma exchange; this combination was indeed associated with the highest recovery rate (77.8%). Anticoagulation, corticosteroids, plasma exchange, and/or IVIg induced a recovery in 69% of cases. While there was no difference between these two combinations, a trend towards statistical significance was observed between each of these regimens compared to all the remaining options (77.8% versus 55.4%, *p* = 0.083 and 69% versus 54.4%, *p* = 0.089). Isolated use of steroids was related to a lower rate of recovery (18.2% versus 58.1% of episodes not treated with corticosteroids; *p* = 0.01) [90]. Plasma exchange is specially indicated when schistocytes are present and should be initiated within 12 hours from the onset [91].

In refractory CAPS, the available evidence comes from anecdotal reports concerning the use of second-line agents as rituximab, eculizumab, and defibrotide.

### 8.2. Rituximab

Rituximab has been used in 20 cases of CAPS, in different combination with anticoagulation, high doses of steroids, plasma exchange, and IVIg. Despite the difficulties in determining the effects of rituximab, a lower mortality compared to larger series emerged [92].

### 8.3. Eculizumab

The complement system, in particular the mediator C5a, has been shown to play a central role in APS. C5a, a potent anaphylatoxic, proinflammatory, and chemotactic molecule, was demonstrated to induce the expression of TF on ECs [93] and neutrophils [94].* In vivo*, C5a was involved in deposition of fibrin in a growing thrombus induced by aPL injection [95]. Eculizumab is a humanized monoclonal antibody which binds to the C5 protein with high affinity, thereby inhibiting its cleavage to C5a and C5b thus preventing the generation of membrane attack complex [96]. To date, eculizumab has been administered to few CAPS patients in whom all the other therapeutic strategies proved to be ineffective. A favourable response was described in two cases [97, 98] and a negative outcome in the others [61]. Eculizumab has also been investigated as a tool to manage APS patients after renal transplantation. In a first report on three consecutive kidney transplant recipients with posttransplant aPL-mediated thrombotic microangiopathy (TMA) resistant to plasmapheresis, treatment with eculizumab improved TMA [99]. In another case series of three patients treated with anticoagulation and eculizumab, no systemic thrombotic events or early graft losses were reported after a follow-up ranging from 4 months to 4 years [100].

### 8.4. Defibrotide

Defibrotide is a polydisperse mixture of 90% single-stranded and 10% double-stranded phosphodiester oligonucleotides derived from the controlled depolymerisation of porcine intestinal mucosal DNA. This pharmacological compound acts by upregulating the release of prostacyclin and prostaglandin E2, reducing concentrations of leukotriene B4, inhibiting monocyte superoxide anion generation, stimulating expression of thrombomodulin in human vascular ECs, and modulating platelet activity [101]. More recently, defibrotide was shown to downregulate TF expression on monocytes [102]. To date, it has been used in two patients with CAPS: in one case this treatment was successful, while the second patient died [103, 104].

## 9. aPL Asymptomatic Carriers

All the studies exploring the thrombotic risk among aPL carriers described a rather low annual rate, around 1% patient-years (range 0–2.8%) [105]. A recent systematic review concluded that this subgroup of patients presents a recurrence rate comparable to aPL negative individuals. This explains why many studies addressing this issue failed to report a clear protective effect for primary prophylaxis and accounts for the evidence that life-long anticoagulation should not be prescribed to those patients not fulfilling laboratory criteria for APS diagnosis.

### 9.1. Low Molecular Weight Heparin

Evidence-based guidelines recommendations strongly advised to administer LMWH thromboprophylaxis to cover high-risk situations such as trauma, infections, surgery, and prolonged immobilization: this was suggested by a 3-year prospective cohort study [41]. Forty-six–seventy-six percent of vascular events in aPL subjects occur concomitantly with other prothrombotic risk factor [11], strongly highlighting the importance of a prompt correction of thrombotic risk factors [23].

### 9.2. Low-Dose Aspirin

A recent meta-analysis showed a decrease in the risk of thrombotic events by LDASA among asymptomatic aPL carriers, SLE patients, and women with obstetrical APS. Noteworthy, such risk reduction did not maintain statistical significance when prospective studies or those with the best methodological quality were considered [106]. Indeed, the only randomized controlled trial included in this meta-analysis, the APLASA study, reported that LDASA was not more effective than placebo for primary prophylaxis of thrombotic events [53]. Consequently, evidence-based guidelines do not recommend universal thromboprophylaxis with LDASA to asymptomatic aPL individuals [23].

Most recently, a randomized controlled trial examining the efficacy of LDASA versus LDASA plus low-intensity anticoagulation as primary thrombotic prevention (ALIWAPAS) was published. In an overall cohort of 166 asymptomatic carriers, the two arms presented the same rate of thrombosis during the 5 years of follow-up, with more bleeding episodes in the LDASA plus warfarin group. These data make the combo therapy not acceptable as a treatment option for these patients [54].

The scenario changes drastically when considering patients with an underlying autoimmune disease. Indeed, there is a growing bulk of evidence that autoimmune diseases represent a thrombotic risk factor* per se*: the inflammation peculiar of these pathologic conditions directly contributes to the accelerated atherosclerosis and the significant cardiovascular mortality observed in these patients [107]. Consequently, given the higher thrombotic rate (around 3.7–4%) reported in this population [12, 51, 52, 108, 109], primary prophylactic treatment is recommended among patients with aPL and an associated autoimmune disease [23]. In this setting, LDASA is the drug most commonly prescribed. The efficacy of LDASA in the primary intervention of aPL positive SLE patients has been investigated in three studies, one retrospective and two prospective, all showing a beneficial effect on thrombosis [12, 108, 109]. In 2000, Wahl and colleagues used a Markov decision analysis model to evaluate the prophylactic role of LDASA in aPL-positive SLE patients, suggesting that it was effective in reducing the number of thrombotic events. In particular, LDASA induced a benefit outweighing the treatment-associated risk of major bleeding [110].

### 9.3. Hydroxychloroquine

An increasing number of experts propose HCQ as primary prophylaxis. Clinical data on the effectiveness of HCQ in preventing aPL-related thrombotic events have been derived from studies in SLE cohorts. Two retrospective studies concordantly showed that the protective effects played by HCQ against thrombosis [111, 112]; a cross-sectional study on 77 APS patients and 56 asymptomatic aPL carriers from a SLE registry proved that the probability of a thrombotic event was decreased by LDASA or HCQ use [50]. In a still unpublished work, Law and coworkers observed a decrease in arterial as well as venous thrombosis in aPL-positive lupus patients receiving HCQ [113]. Accordingly, a systematic review concluded that antimalarials exert an antithrombotic action among SLE patients [114].

Details of the studies investigating management of aPL asymptomatic carriers are listed in Table 3.

## 10. Potential Future Therapeutic Target

To date, management strategies in APS have been mainly restricted to anticoagulation, which is not effective in all patients. Hopefully, the unravelling of APS pathogenic mechanisms may allow identifying alternative therapeutic targets.

### 10.1. Novel Molecules Blocking *β*2/Anti-*β*2GPI Antibody Binding

(i) TIFI is a 20-amino acid synthetic peptide that spans Thr101–Thr120 of ULB0-HCMVA from human cytomegalovirus, which shares similarities with the PL-binding site in *β*2GPI molecule, DV. TIFI is not targeted by aPL;* in vitro* evidence suggests that TIFI inhibits the binding of labelled *β*2GPI to human ECs and mouse monocytes [115]. These findings were also confirmed in animal models: the infusion of this synthetic peptide inhibited aPL-mediated thrombosis by decreasing the thrombus size produced in response to aPL and by reducing the binding of fluoresceinated *β*2GPI to ECs [116].

(ii) Accordingly, a synthetic *β*2GPI-DI was shown to inhibit aPL-mediated prothrombic effects both* in vivo* and* in vitro* [117].

(iii) MBB2 is a novel single chain variable fragment- (scFv-) Fc monoclonal antibody targeting DI of human, rat, and mouse *β*2GPI. When infused to experimental animals, MBB2 caused blood clots in rat mesenteric microcirculation after LPS priming. A noncomplement-fixing variant of MBB2, MBB2ΔCH2, has also been developed. MBB2ΔCH2 displays the same antigen specificity of MBB2 but, lacking the CH2 domain, is unable to activate the complement cascade. MBB2ΔCH2 has been shown to prevent the aPL-coagulant effects* in vivo* by competing with circulating aPL for binding to *β*2GPI.* In vivo*, the CH2-deleted monoclonal antibody significantly reduced mesenteric thrombus formation and vessel occlusion [118].

(iv) It can also be postulated that antagonists of the receptors involved in *β*2GPI cell binding may exert a therapeutic potential [119]. The use of antagonists or neutralizing monoclonal antibodies acting on TLR2/4 might be speculated in APS. In addition, DV of *β*2GPI binds the A1 ligand-binding type A module of ApoER2′, a dimer composed of two A1 molecules joined by a flexible linker, has been shown to inhibit anti-*β*2GPI antibody/dimerized *β*2GPI immune complexes from binding negatively charged PL and ApoER2′* in vitro*, more potently than A1 in the monomeric form [120]. More recently, proofs of the effectiveness of this dimeric molecule were obtained* in vivo*, in two animal models of APS. Indeed, treatment with A1-A1 efficiently reduced thrombus size* in vivo* in the presence of chronic autoimmune anti-*β*2GPI antibody in lupus-prone (NZW3BXSB)F1 male mice as well as in wild-type mice after infusion with anti-*β*2GPI antibodies [121].

(v) Similarly, blockers of the intracellular mediators involved in aPL-activated signalling pathways may reverse the prothrombotic phenotype: NF*κ*B and p38MAPK inhibitors have been shown to be effective in preventing aPL mediated prothrombotic and proinflammatory effects* in vitro* [122]. More recently, the NF*κ*B inhibitor DHMEQ was demonstrated to ameliorate the prothrombotic state induced by the infusion of the monoclonal antibody WB-6 in normal BALB/c mice [123].

### 10.2. Novel Molecules Interfering with aPL-Induced Mediators

(i) It can be speculated that TF inhibition may prevent thrombosis in APS [124]. Currently, there are few drugs available on the market blocking TF expression: ACE inhibitors, dilazep, defibrotide, and dipyridamole. In particular, both dilazep and dipyridamole have been shown to block the upregulation of TF specifically induced by polyclonal IgG purified from APS patients in monocytes [125, 126]. However, their role in APS management has been scarcely documented.

(ii) aPL also upregulate GPIIb/IIIa thus leading to platelet aggregation. Abciximab is a specific GPIIb/IIIa inhibitor routinely prescribed in stroke and acute coronary syndromes, which might be beneficial in APS [127].

(iii) Protein disulphide isomerase is the enzyme responsible for the formation of two disulphide bridges within *β*2GPI molecule, a reaction leading to an oxidized and immunogenic molecule. This enzyme is inhibited by quercetin-3-rutinoside, whose potential pharmacological effect in APS has to be investigated. In animal models, inhibitors of PDI were effective in treating thrombosis [128].

(iv) Given that oxidation leads to the unmasking of the critical B-cell epitope, it might be worth exploring the role of antioxidant compounds as N-acetylcysteine, vitamin C, and coenzyme Q10 in APS [120]. In an* in vitro* study, the inhibition of intracellular reactive oxygen species in monocytes prevented the upregulation of TF induced by aPL [129].

(v) Similarly, TNF-*α* and IL-6 are proinflammatory mediators induced by aPL: it is therefore reasonable to hypothesize that the blockade of these cytokines with biologic agents may be clinically beneficial [122].

**Figure 1:**
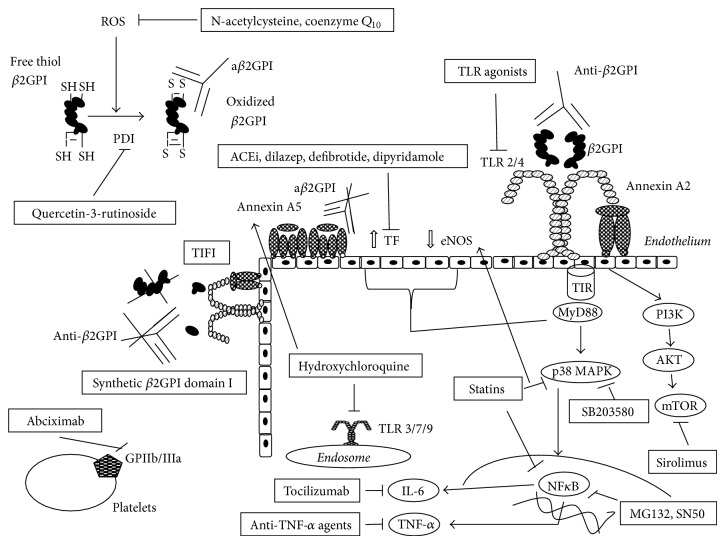
Mechanisms of action of potential future therapeutic tools in APS. ROS: reactive oxygen species; *β*2GPI: *β*2 glycoprotein I; a*β*2GPI: antibodies against *β*2GPI; PDI: protein disulfide isomerase; ACEi: angiotensin-converting enzyme inhibitors; TLR: Toll-like receptor; TF: tissue factor; eNOS: endothelial nitric oxide synthase; GP: glycoprotein; IL: interleukin; TNF: tumour necrosis factor; PI3K: phosphatidylinositol 3-kinase; mTOR: mammalian target of rapamycin.

The mechanisms of action of the potential future therapeutic tools in APS management are detailed in Figure 1.

## 11. Conclusions

Antiplatelet and anticoagulant agents still provide the cornerstone of APS treatment, even though many clinical issues remain still unresolved. Hopefully, these critical issues will be soon overcome thanks to large, multicentre clinical trials. A well-designed study should account for the relative contribution of the different clinical variables: APS manifestations may be indeed highly diverse, being related to aPL profile as well as to the concomitant cardiovascular risk factors.

**Figure 2:**
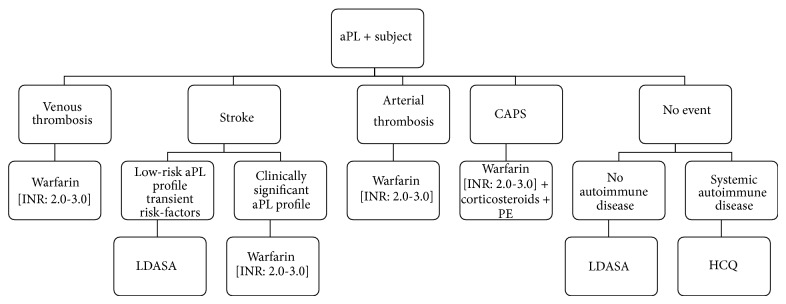
Flow-diagram of the therapeutic approach to thrombotic APS. aPL: antiphospholipid antibodies; CAPS: catastrophic antiphospholipid syndrome; LDASA: low-dose aspirin; PE: plasma exchange; HCQ: hydroxychloroquine.

For the time being, an evidence-based approach would be the following (Figure 2):

- patients with venous events should receive long-term anticoagulation at an INR target of 2.0-3.0;
- patients with a history of arterial thrombosis should receive long-term anticoagulation at an INR target of 2.0-3.0;
- subjects who develop a stroke and present a low-risk aPL profile without any associated autoimmune condition may be prescribed with LDASA;
- stroke patients at higher thrombotic risk and individuals with a history of arterial thrombosis should receive long term anticoagulation;
- a primary thromboprophylaxis with LDASA should be instituted in asymptomatic carriers with a clinically significant aPL profile or additional thrombotic risk factors; HCQ should be prescribed to subjects with underlying autoimmune diseases.

In addition, a strict management of prothrombotic risk factors is warranted in all aPL carriers; unfortunately, no study has yet addressed the potential effects of controlling cardiovascular status on outcome. Similarly, the pleiotropic effects of agents such as statins and HCQ should be further assessed: the addition of these drugs to standard anticoagulation may lead to a better disease control. Moreover, the identification of novel diagnostic tools, such as antibodies against DI of *β*2GPI or against phosphatidylserine/prothrombin, may allow more precise risk stratification, leading to a tailored treatment strategy. Research has been galvanized to identify novel therapeutic targets: hopefully, such pharmacological agents might revolutionize APS management in the near future.

## Figures and Tables

**Table 1:** Details of clinical studies investigating efficacy of antiplatelet and anticoagulant regimens in thrombotic APS.

Author, year [Ref]	Type of study	*N* patients	Sapporo criteria	Thrombotic events A/V	Treatments	Observation time	Recurrence rate	Main findings
Rosove and Brewer, 1992 [33]	RC	70	No	31/39	None LDASA Warfarin INR <2.0 Warfarin INR 2.0–2.9 Warfarin INR >3.0	161.2 pt.-years 37.8 pt.-years 11.3 pt.-years 40.9 pt.-years 110.2 pt.-years	0.19/pt.-year 0.32/pt.-year 0.57/pt.-year 0.07/pt.-year 0	Intermediate-high intensity warfarin conferred better antithrombotic protection than low-intermediate warfarin and LDASA

Derksen et al., 1993 [35]	RC	19	Yes	0/19	None Warfarin INR 2.5–4.0	8–248 months	NA	Anticoagulation was effective in preventing thrombosis compared to placebo

Khamashta et al., 1995 [34]	RC	147	Yes	67/80	None LDASA Warfarin INR <3.0 (+LDASA) Warfarin INR >3.0 (+LDASA)	280.6 pt.-years 240.3 pt.-years 141.3 pt.-years 197.3 pt.-years	0.29/pt.-year 0.18/pt.-year 0.23/pt.-year 0.015/pt.-year	Warfarin INR >3.0 ± LDASA was significantly more effective than warfarin INR <3.0 ± LDASA in preventing thrombotic recurrences

Krnic-Barrie et al., 1997 [15]	RC	61	No	38/23	None LDASA Warfarin Warfarin + LDASA	124.9 pt.-years 36.6 pt.-years 63.0 pt.-years 30.6 pt.-years	A: 0.192/V: 0.11/pt.-year A: 0.082/V: 0.027/pt.-year A: 0.048/V: 0/pt.-year A: 0/V: 0/pt.-year	Warfarin treatment, with or without LDASA, was more effective than placebo and LDASA alone in preventing recurrence

Munoz-Rodríguez et al., 1999 [40]	RC	47	Yes	19/28	None LDASA Warfarin INR 2.5–3.5	4–50 months	91% 41% 19%	Warfarin was more effective than placebo and LDASA in preventing thrombotic recurrences

Ruiz-Irastorza et al., 2002 [14]	RC	66	Yes	51/32	Warfarin INR 3.0–4.0	66 pt.-years	0.09/pt.-year	Despite high-intensity warfarin, the risk of thrombotic recurrences was high

Wittkowsky et al., 2006 [39]	RC	36	Yes	14/16	Warfarin INR 2.0–3.0 Warfarin INR >3.0	62.5 pt.-years	0.096/pt.-year	67% of the recurrences occurred at INR <3.0

Girón-González et al., 2004 [41]	PC	158	Yes	70/106	Warfarin INR 2.5–3.5	624 pt.-years	0.005/pt.-year	Thrombotic recurrence was associated with INR below target

Ames et al., 2005 [27]	PC	67	Yes	17/50	Warfarin INR <2.0 Warfarin INR 2.0–3.0 Warfarin INR 3.1–4.0 Warfarin INR >4.0	9 weeks 122 weeks 9 weeks 5 weeks	0 0.04/pt.-year 0.1/pt.-year 0	Recurrence rates were higher in patients receiving high-intensity than low-intensity anticoagulation

Ginsberg et al., 1995 [30]	PC subgroup analysis	16	No	0/16	None Warfarin INR 2.0–3.0	8.7 months 3 months	18% 0	No recurrence was observed with warfarin INR 2.0–3.0

Prandoni et al., 1996 [29]	RC subgroup analysis	15	Yes	0/15	None Warfarin INR 2.0–3.0	1–10 years	0.038/pt.-year 0	No recurrence was observed with warfarin INR 2.0–3.0

Rance et al., 1997 [31]	RC subgroup analysis	27	No	0/27	None Warfarin INR 2.0–3.0	1–4 years	NA 0	No recurrence was observed with warfarin INR 2.0–3.0

Schulman et al., 1998 [32]	RCT subgroup analysis	68	No	0/68	None Warfarin INR 2.0–2.8	4 years	0.01/pt.-year 0	No recurrence was observed with warfarin INR 2.0–2.8

Levine et al., 2004 [42]	RCT subgroup analysis	720	No	720/0	ASA Warfarin INR 1.4–2.8	2 years	22.18% 26.15%	ASA and low-intensity anticoagulation were equally effective for secondary stroke prevention

Crowther et al., 2003 [19]	RCT	114	Yes	27/87	Warfarin INR 2.0–3.0 Warfarin INR 3.1–4.0	2.7 years	0.013/pt.-year 0.032/pt.-year	Warfarin at INR 2.0–3.0 was as effective as warfarin at INR 3.1–4.0 in secondary prevention of thrombosis

Finazzi et al., 2005 [20]	RCT	109	Yes	44/75	Warfarin INR 2.0–3.0 Warfarin INR 3.0–4.5	3.3 years 3.5 years	0.016/pt.-year 0.031/pt.-year	Warfarin at INR 2.0–3.0 was as effective as warfarin at INR 3.1–4.5 in secondary prevention of thrombosis

Tan et al., 2009 [16]	RC	59	Yes	29/30	Warfarin INR 2.0–3.0 Warfarin INR 3.1–4.0	8.8 years	23.7%	Warfarin at INR 2.0–3.0 is as effective as warfarin at INR >3 in preventing venous rethromboses and less effective in preventing arterial rethromboses

Cervera et al., 2009 [2]	PC	1000	Yes		LDASA Warfarin	5 years	14% 21.4%	Antiaggregation and anticoagulation are associated with a significant recurrence rate

Pengo et al., 2010 [18]	RC	160	Yes	76/69	No treatment Warfarin (median INR 2.3)	10 years	44.2%	Oral anticoagulation was the only predictor of thromboembolic events

Okuma et al., 2010 [43]	RCT	20	No	0/20	LDASA Warfarin INR 2.0–3.0 + LDASA	3.9 years	NA	Warfarin + LDASA was more effective that LDASA alone in secondary prevention of stroke

Fujieda et al., 2012 [44]	RC	82	Yes	82/0	Warfarin Antiplatelet Warfarin + antiplatelet Dual antiplatelet	8.5 years	3.4/100/pt.-year 0	Dual antiplatelet regimen was beneficial in preventing recurrences in refractory APS

A: arterial; V: venous; PC: prospective cohort; RC: retrospective cohort; RCT: randomized controlled trial; LDASA: low-dose aspirin.

**Table 2:** Details of clinical studies investigating efficacy of long-term treatment with LMWH in resistant thrombotic APS.

Author, year [Ref]	Type of study	*N* patients	Inclusion criteria	Treatments	Observation time	Recurrence Rate
Bick and Rice, 1999 [45]	Retrospective	24	APS patients resistant or intolerant to warfarin	Dalteparin	309 days	0

Ahmed et al., 2002 [46]	Case-report	1	APS patient with difficulties in keeping INR in target	Enoxaparin 1.5 mg/kg daily	90 days	1 (pulmonary TE)

Dentali et al., 2005 [47]	Case-report	2	APS patients refractory to warfarin	Enoxaparin 10000 IU td Dalteparin 10000 U td	2 years 6 years	0

Vargas-Hitos et al., 2011 [48]	Retrospective	23	APS patients refractory to warfarin	Enoxaparin	36 months	0.13

**Table 3:** Details of clinical studies investigating optimal management of aPL carriers.

Author, year [Ref]	Type of study	*N* patients	Inclusion criteria	Treatment	Observation time	Event Rate	Main findings
Erkan et al., 2001 [49]	Retrospective	65	aPL+ women with 1 foetal loss	LDASA No treatment	Mean 8.1 years	10% 59%	LDASA beneficial in aPL+ women with 1 foetal loss

Erkan et al., 2002 [50]	Cross-sectional	56	aPL+ asymptomatic subjects	LDASA/HCQ	6 months	0	LDASA/HCQ beneficial in asymptomatic aPL+ individuals

Girón-González et al., 2004 [41]	Prospective	178	aPL+ asymptomatic subjects	LMWH/LDASA prophylaxis during high-risk situation	3 years	0	LMWH/LDASA prophylaxis during high-risk situation beneficial as primary prophylaxis in aPL carriers

Mok et al., 2005 [51]	Retrospective	272	SLE patients (aPL+ 29%)	HCQ No HCQ	27 years	1.26/100 patient-years	Patients taking HCQ had fewer thrombotic complications than those who were not (OR 0.17, 95% CI 0.07–0.44; *p* < 0.0001)

Tarr et al., 2007 [52]	Prospective	81	aPL+ SLE patients	LDASA None	5 years	1.9% 6.9%	LDASA/HCQ beneficial in asymptomatic aPL+ SLE patients

Erkan et al., 2007 [53]	Randomized, controlled	98	aPL positive subjects	LDASA No treatment	Mean 2.46 ± 0.76 years	2.75/100 patient-years 0/100 patient-years	LDASA not beneficial in the primary prophylaxis of aPL positive carriers

Tektonidou et al., 2009 [12]	Prospective	288	144 aPL+ SLE patients 144 aPL− SLE patients	LDASA/HCQ	104 months 112 months	20.1% 7.6%	LDASA and HCQ protective against thrombosis in SLE patients

Cuadrado et al., 2014 [54]	RCT	166	aPL+ patients	LDASA LDASA + low intensity warfarin	5 years	4.9% 4.8%	LDASA and LDASA + warfarin were equally effective, but lower bleeding risk with LDASA only

## References

[1] Miyakis S., Lockshin M. D., Atsumi T. (2006). International consensus statement on an update of the classification criteria for definite antiphospholipid syndrome (APS). *Journal of Thrombosis and Haemostasis*.

[2] Cervera R., Khamashta M. A., Shoenfeld Y. (2009). Morbidity and mortality in the antiphospholipid syndrome during a 5-year period: a multicentre prospective study of 1000 patients. *Annals of the Rheumatic Diseases*.

[3] Meroni P. L., Chighizola C. B., Rovelli F., Gerosa M. (2014). Antiphospholipid syndrome in 2014: more clinical manifestations, novel pathogenic players and emerging biomarkers. *Arthritis Research and Therapy*.

[4] Cervera R., Piette J.-C., Font J. (2002). Antiphospholipid syndrome: clinical and immunologic manifestations and patterns of disease expression in a cohort of 1,000 patients. *Arthritis and Rheumatism*.

[5] Chighizola C. B., Gerosa M., Meroni P. L. (2014). New tests to detect antiphospholipid antibodies: anti-domain i beta-2-glycoprotein-I antibodies. *Current Rheumatology Reports*.

[6] Meroni P. L., Borghi M. O., Raschi E., Tedesco F. (2011). Pathogenesis of antiphospholipid syndrome: understanding the antibodies. *Nature Reviews Rheumatology*.

[7] Canaud G., Bienaimé F., Tabarin F. (2014). Inhibition of the mTORC pathway in the Antiphospholipid syndrome. *The New England Journal of Medicine*.

[8] Andreoli L., Chighizola C. B., Banzato A., Pons-Estel G. J., de Jesus G. R., Erkan D. (2013). Estimated frequency of antiphospholipid antibodies in patients with pregnancy morbidity, stroke, myocardial infarction, and deep vein thrombosis: a critical review of the literature. *Arthritis Care & Research*.

[9] Chighizola C. B., Andreoli L., Banzato A. (2013). The association between antiphospholipid antibodies and related clinical outcomes: a critical review of the literature. *Arthritis & Rheumatism*.

[10] De Groot P. G., Lutters B., Derksen R. H. W. M., Lisman T., Meijers J. C. M., Rosendaal F. R. (2005). Lupus anticoagulants and the risk of a first episode of deep venous thrombosis. *Journal of Thrombosis and Haemostasis*.

[11] Ruffatti A., Del Ross T., Ciprian M. (2009). Risk factors for a first thrombotic event in antiphospholipid antibody carriers. A multicentre, retrospective follow-up study. *Annals of the Rheumatic Diseases*.

[12] Tektonidou M. G., Laskari K., Panagiotakos D. B., Moutsopoulos H. M. (2009). Risk factors for thrombosis and primary thrombosis prevention in patients with systemic lupus erythematosus with or without antiphospholipid antibodies. *Arthritis Care and Research*.

[13] Shoenfeld Y., Blank M., Cervera R., Font J., Raschi E., Meroni P.-L. (2006). Infectious origin of the antiphospholipid syndrome. *Annals of the Rheumatic Diseases*.

[14] Ruiz-Irastorza G., Khamashta M. A., Hunt B. J., Escudero A., Cuadrado M. J., Hughes G. R. V. (2002). Bleeding and recurrent thrombosis in definite antiphospholipid syndrome: analysis of a series of 66 patients treated with oral anticoagulation to a target international normalized ratio of 3.5. *Archives of Internal Medicine*.

[15] Krnic-Barrie S., O'Connor C. R., Looney S. W., Pierangdi S. S., Harris E. N. (1997). A retrospective review of 61 patients with antiphospholipid syndrome: analysis of factors influencing recurrent thrombosis. *Archives of Internal Medicine*.

[16] Tan B. E., Thong B. Y. H., Shivananda S., Han W. W., Chng H. H. (2009). Clinical manifestations and outcomes of antithrombotic treatment of the Tan Tock Seng Hospital Singapore antiphospholipid syndrome cohort. *Lupus*.

[17] Neville C., Rauch J., Kassis J. (2009). Antiphospholipid antibodies predict imminent vascular events independently from other risk factors in a prospective cohort. *Thrombosis and Haemostasis*.

[18] Pengo V., Ruffatti A., Legnani C. (2010). Clinical course of high-risk patients diagnosed with antiphospholipid syndrome. *Journal of Thrombosis and Haemostasis*.

[19] Crowther M. A., Ginsberg J. S., Julian J. (2003). A comparison of two intensities of warfarin for the prevention of recurrent thrombosis in patients with the antiphospholipid antibody syndrome. *The New England Journal of Medicine*.

[20] Finazzi G., Marchioli R., Brancaccio V. (2005). A randomized clinical trial of high-intensity warfarin vs. conventional antithrombotic therapy for the prevention of recurrent thrombosis in patients with the antiphospholipid syndrome (WAPS). *Journal of Thrombosis and Haemostasis*.

[21] Lim W., Crowther M. A., Eikelboom J. W. (2006). Management of antiphospholipid antibody syndrome: a systematic review. *Journal of the American Medical Association*.

[22] Ruiz-Irastorza G., Hunt B. J., Khamashta M. A. (2007). A systematic review of secondary thromboprophylaxis in patients with antiphospholipid antibodies. *Arthritis Care and Research*.

[23] Ruiz-Irastorza G., Cuadrado M. J., Ruiz-Arruza I. (2011). Evidence-based recommendations for the prevention and long-term management of thrombosis in antiphospholipid antibody-positive patients: report of a task force at the 13th International Congress on Antiphospholipid Antibodies. *Lupus*.

[24] Cohen H., MacHin S. J. (2010). Antithrombotic treatment failures in antiphospholipid syndrome: the new anticoagulants?. *Lupus*.

[25] Guerin J., Sheng Y., Reddel S., Iverson G. M., Chapman M. G., Krilis S. A. (2002). Heparin inhibits the binding of *β*2-glycoprotein I to phospholipids and promotes the plasmin-mediated inactivation of this blood protein. Elucidation of the consequences of the two biological events in patients with the anti-phospholipid syndrome. *The Journal of Biological Chemistry*.

[26] Bazzan M., Vaccarino A., Stella S. (2013). Thrombotic recurrences and bleeding events in APS vascular patients: a review from the literature and a comparison with the APS Piedmont Cohort. *Autoimmunity Reviews*.

[27] Ames P. R. J., Ciampa A., Margaglione M., Scenna G., Iannaccone L., Brancaccio V. (2005). Bleeding and re-thrombosis in primary antiphospholipid syndrome on oral anticoagulation: an 8-year longitudinal comparison with mitral valve replacement and inherited thrombophilia. *Thrombosis and Haemostasis*.

[28] Kearon C., Akl E. A., Comerota A. J. (2012). Antithrombotic therapy for wi disease: Antithrombotic Therapy and Prevention of Thrombosis, 9th ed: American College of Chest Physicians Evidence-Based Clinical Practice Guidelines. *Chest*.

[29] Prandoni P., Simioni P., Girolami A. (1996). Antiphospholipid antibodies, recurrent thromboembolism, and intensity of warfarin anticoagulation. *Thrombosis and Haemostasis*.

[30] Ginsberg J. S., Wells P. S., Brill-Edwards P. (1995). Antiphospholipid antibodies and venous thromboembolism. *Blood*.

[31] Rance A., Emmerich J., Fiessinger J.-N. (1997). Anticardiolipin antibodies and recurrent thromboembolism. *Thrombosis and Haemostasis*.

[32] Schulman S., Svenungsson E., Granqvist S. (1998). Anticardiolipin antibodies predict early recurrence of thromboembolism and death among patients with venous thromboembolism following anticoagulant therapy. Duration of Anticoagulation Study Group. *American Journal of Medicine*.

[33] Rosove M. H., Brewer P. M. C. (1992). Antiphospholipid thrombosis: clinical course after the first thrombotic event in 70 patients. *Annals of Internal Medicine*.

[34] Khamashta M. A., Cuadrado M. J., Mujic F., Taub N. A., Hunt B. J., Hughes G. R. V. (1995). The management of thrombosis in the antiphospholipid-antibody syndrome. *The New England Journal of Medicine*.

[35] Derksen R. H. W. M., de Groot P. G., Kater L., Nieuwenhuis H. K. (1993). Patients with antiphospholipid antibodies and venous thrombosis should receive long term anticoagulant treatment. *Annals of the Rheumatic Diseases*.

[36] Michaels L. (1971). Incidence of thromboembolism after stopping anticoagulant therapy. Relationship to hemorrhage at the time of termination. *Journal of the American Medical Association*.

[37] Coloma Bazán E., Donate López C., Moreno Lozano P., Cervera R., Espinosa G. (2013). Discontinuation of anticoagulation or antiaggregation treatment may be safe in patients with primary antiphospholipid syndrome when antiphospholipid antibodies became persistently negative. *Immunologic Research*.

[38] Criado-García J., Fernández-Puebla R. A., López Jiménez L., Velasco F., Santamaría M., Blanco-Molina A. (2008). Anticoagulation treatment withdrawal in primary antiphospholipid syndrome when anticardiolipin antibodies become negative. *Revista Clinica Espanola*.

[39] Wittkowsky A. K., Downing J., Blackburn J., Nutescu E. (2006). Warfarin-related outcomes in patients with antiphospholipid antibody syndrome managed in an anticoagulation clinic. *Thrombosis and Haemostasis*.

[40] Muñoz-Rodríguez F. J., Font J., Cervera R. (1999). Clinical study and follow-up of 100 patients with the antiphospholipid syndrome. *Seminars in Arthritis and Rheumatism*.

[41] Girón-González J. A., del Río E. G., Rodríguez C., Rodríguez-Martorell J., Serrano A. (2004). Antiphospholipid syndrome and asymptomatic carriers of antiphospholipid antibody: prospective analysis of 404 individuals. *The Journal of Rheumatology*.

[42] Levine S. R., Brey R. L., Tilley B. C. (2004). Antiphospholipid antibodies and subsequent thrombo-occlusive events in patients with ischemic stroke. *The Journal of the American Medical Association*.

[43] Okuma H., Kitagawa Y., Yasuda T., Tokuoka K., Takagi S. (2010). Comparison between single antiplatelet therapy and combination of antiplatelet and anticoagulation therapy for secondary prevention in ischemic stroke patients with antiphospholipid syndrome. *International Journal of Medical Sciences*.

[44] Fujieda Y., Amengual O., Watanabe T. (2012). Dual antiplatelet therapy as prophylaxis of recurrent arterial thrombosis in patients with antiphospholipid syndrome. *Arthritis & Rheumatology*.

[45] Bick R. L., Rice J. (1999). Long-term outpatient dalteparin (fragmin) therapy for arterial and venous thrombosis: efficacy and safety—a preliminary report. *Clinical and Applied Thrombosis/Hemostasis*.

[46] Ahmed S., Karim A., Patel D., Siddiqui R., Mattana J. (2002). Low-molecular weight heparin: treatment failure in a patient with primary antiphospholipid antibody syndrome. *American Journal of the Medical Sciences*.

[47] Dentali F., Manfredi E., Crowther M., Ageno W. (2005). Long-duration therapy with low molecular weight heparin in patients with antiphospholipid antibody syndrome resistant to warfarin therapy. *Journal of Thrombosis and Haemostasis*.

[48] Vargas-Hitos J. A., Ateka-Barrutia O., Sangle S., Khamashta M. A. (2011). Efficacy and safety of long-term low molecular weight heparin in patients with antiphospholipid syndrome. *Annals of the Rheumatic Diseases*.

[49] Erkan D., Merrill J. T., Yazici Y., Sammaritano L., Buyon J. P., Lockshin M. D. (2001). High thrombosis rate after fetal loss in antiphospholipid syndrome: effective prophylaxis with aspirin. *Arthritis and Rheumatism*.

[50] Erkan D., Yazici Y., Peterson M. G., Sammaritano L., Lockshin M. D. (2002). A cross-sectional study of clinical thrombotic risk factors and preventive treatments in antiphospholipid syndrome. *Rheumatology*.

[51] Mok Y. M., Chan E. Y. T., Fong D. Y. T., Leung K. F. S., Woon S. W., Chak S. L. (2005). Antiphospholipid antibody profiles and their clinical associations in Chinese patients with systemic lupus erythematosus. *Journal of Rheumatology*.

[52] Tarr T., Lakos G., Bhattoa H. P., Shoenfeld Y., Szegedi G., Kiss E. (2007). Analysis of risk factors for the development of thrombotic complications in antiphospholipid antibody positive lupus patients. *Lupus*.

[53] Erkan D., Harrison M. J., Levy R. (2007). Aspirin for primary thrombosis prevention in the antiphospholipid syndrome: a randomized, double-blind, placebo-controlled trial in asymptomatic antiphospholipid antibody-positive individuals. *Arthritis and Rheumatism*.

[54] Cuadrado M. J., Bertolaccini M. L., Seed P. T. (2014). Low-dose aspirin vs low-dose aspirin plus low-intensity warfarin in thromboprophylaxis: a prospective, multicentre, randomized, open, controlled trial in patients positive for antiphospholipid antibodies (ALIWAPAS). *Rheumatology*.

[55] Kužnik A., Benčina M., Švajger U., Jeras M., Rozman B., Jerala R. (2011). Mechanism of endosomal TLR inhibition by antimalarial drugs and imidazoquinolines. *Journal of Immunology*.

[56] Espinola R. G., Pierangeli S. S., Ghara A. E., Harris E. N. (2002). Hydroxychloroquine reverses platelet activation induced by human IgG antiphospholipid antibodies. *Thrombosis and Haemostasis*.

[57] Rand J. H., Wu X.-X., Quinn A. S., Chen P. P., Hathcock J. J., Taatjes D. J. (2008). Hydroxychloroquine directly reduces the bindin of antiphospholipid antibody-*β*2-glycoprotein I complexes to phospholipid bilayers. *Blood*.

[58] Rand J. H., Wu X.-X., Quinn A. S. (2010). Hydroxychloroquine protects the annexinA5 anticoagulant shield from disruption by antiphospholipid antibodies: evidence for a novel effect for an old antimalarial drug. *Blood*.

[59] Edwards M. H., Pierangeli S., Liu X., Barker J. H., Anderson G., Nigel Harris E. (1997). Hydroxychloroquine reverses thrombogenic properties of antiphospholipid antibodies in mice. *Circulation*.

[60] Schmidt-Tanguy A., Voswinkel J., Henrion D. (2013). Antithrombotic effects of hydroxychloroquine in primary antiphospholipid syndrome patients. *Journal of Thrombosis and Haemostasis*.

[61] Erkan D., Aguiar C. L., Andrade D. (2014). 14th International Congress on Antiphospholipid Antibodies: task force report on antiphospholipid syndrome treatment trends. *Autoimmunity Reviews*.

[62] Chighizola C. B., Moia M., Meroni P. L. (2014). New oral anticoagulants in thrombotic antiphospholipid syndrome. *Lupus*.

[63] Noel N., Dutasta F., Costedoat-Chalumeau N. (2014). Safety and efficacy of new oral direct inhibitors of thrombin and factor Xa in antiphospholipid syndrome. *Arthritis & Rheumatism*.

[64] Sciascia S., Hunt B. (2014). Rivaroxaban use in patients with antiphospholipid syndrome patients and previous poor anticoagulation control with vitamin K antagonists. *Arthritis & Rheumatology*.

[65] Schaefer J. K., McBane R. D., Black D. F., Williams L. N., Moder K. G., Wysokinski W. E. (2014). Failure of dabigatran and rivaroxaban to prevent thromboembolism in antiphospholipid syndrome: a case series of three patients. *Thrombosis and Haemostasis*.

[66] Medina G., Gutiérrez-Moreno A. L., Vera-Lastra O., Saavedra M. A., Jara L. J. (2011). Prevalence of metabolic syndrome in primary antiphospholipid syndrome patients. *Autoimmunity Reviews*.

[67] Ferrara D. E., Swerlick R., Casper K. (2004). Fluvastatin inhibits up-regulation of tissue factor expression by antiphospholipid antibodies on endothelial cells. *Journal of Thrombosis and Haemostasis*.

[68] Meroni P. L., Raschi E., Testoni C. (2001). Statins prevent endothelial cell activation induced by antiphospholipid (anti-*β*2-glycoprotein I) antibodies: effect on the proadhesive and proinflammatory phenotype. *Arthritis & Rheumatism*.

[69] Martínez-Martínez L. A., Amigo M. C., Orozco A. (2006). Effect of rosuvastatin on vascular cell adhesion molecule 1 (vcam-1) expression by human endothelial cells exposed to antiphospholipid syndrome serum. *Journal of Clinical Rheumatology*.

[70] López-Pedrera C., Ruiz-Limón P., Aguirre M. Á. (2011). Global effects of fluvastatin on the prothrombotic status of patients with antiphospholipid syndrome. *Annals of the Rheumatic Diseases*.

[71] Erkan D., Willis R., Murthy V. L. (2014). A prospective open-label pilot study of fluvastatin on proinflammatory and prothrombotic biomarkers in antiphospholipid antibody positive patients. *Annals of the Rheumatic Diseases*.

[72] Agmon-Levin N., Blank M., Zandman-Goddard G. (2011). Vitamin D: an instrumental factor in the anti-phospholipid syndrome by inhibition of tissue factor expression. *Annals of the Rheumatic Diseases*.

[73] Piantoni S., Andreoli L., Allegri F., Meroni P. L., Tincani A. (2012). Low levels of vitamin D are common in primary antiphospholipid syndrome with thrombotic disease. *Reumatismo*.

[74] Klack K., Carvalho J. F. D. (2010). High frequency of vitamin D insufficiency in primary antiphospolipid syndrome. *Joint Bone Spine*.

[75] Paupitz J. A., de Carvalho J. F., Caparbo V. F., Klack K., Pereira R. M. R. (2010). Primary antiphospholipid syndrome in premenopausal women: low vitamin D, high fat mass and maintained bone mineral mass. *Lupus*.

[76] Andreoli L., Piantoni S., Dall'Ara F., Allegri F., Meroni P. L., Tincani A. (2012). Vitamin D and antiphospholipid syndrome. *Lupus*.

[77] Lee A. Y. Y., Levine M. N., Baker R. I. (2003). Low-molecular-weight heparin versus a coumarin for the prevention of recurrent venous thromboembolism in patients with cancer. *The New England Journal of Medicine*.

[78] Konova E. (2005). Intravenous immunoglobulin therapy in antiphospholipid syndrome. *Clinical Reviews in Allergy and Immunology*.

[79] Pierangeli S. S., Espinola R., Liu X., Nigel Harris E., Salmon J. E. (2001). Identification of an Fc*γ* receptor-independent mechanism by which intravenous immunoglobulin ameliorates antiphospholipid antibody-induced thrombogenic phenotype. *Arthritis and Rheumatism*.

[80] Sherer Y., Levy Y., Shoenfeld Y. (2000). Intravenous immunoglobulin therapy of antiphospholipid syndrome. *Rheumatology*.

[81] Tenti S., Guidelli G. M., Bellisai F., Galeazzi M., Fioravanti A. (2013). Long-term treatment of antiphospholipid syndrome with intravenous immunoglobulin in addition to conventional therapy. *Clinical & Experimental Rheumatology*.

[82] Sciascia S., Giachino O., Roccatello D. (2012). Prevention of thrombosis relapse in antiphospholipid syndrome patients refractory to conventional therapy using intravenous immunoglobulin. *Clinical and Experimental Rheumatology*.

[83] Kahn P., Ramanujam M., Bethunaickan R. (2008). Prevention of murine antiphospholipid syndrome by BAFF blockade. *Arthritis & Rheumatism*.

[84] Akkerman A., Huang W., Wang X. (2004). CTLA4Ig prevents initiation but not evolution of anti-phospholipid syndrome in NZW/BXSB mice. *Autoimmunity*.

[85] Stohl W., Hiepe F., Latinis K. M. (2012). Belimumab reduces autoantibodies, normalizes low complement levels, and reduces select B cell populations in patients with systemic lupus erythematosus. *Arthritis and Rheumatism*.

[86] Ramos-Casals M., Brito-Zerón P., Muñoz S., Soto M.-J. (2008). A systematic review of the off-label use of biological therapies in systemic autoimmune diseases. *Medicine*.

[87] Khattri S., Zandman-Goddard G., Peeva E. (2012). B-cell directed therapies in antiphospholipid antibody syndrome—new directions based on murine and human data. *Autoimmunity Reviews*.

[88] Erkan D., Vega J., Ramón G., Kozora E., Lockshin M. D. (2013). A pilot open-label phase II trial of rituximab for non-criteria manifestations of antiphospholipid syndrome. *Arthritis & Rheumatism*.

[89] Suzuki K., Nagasawa H., Kameda H. (2009). Severe acute thrombotic exacerbation in two cases with anti-phospholipid syndrome after retreatment with rituximab in phase I/II clinical trial for refractory systemic lupus erythematosus. *Rheumatology*.

[90] Bucciarelli S., Espinosa G., Cervera R. (2006). Mortality in the catastrophic antiphospholipid syndrome: causes of death and prognostic factors in a series of 250 patients. *Arthritis and Rheumatism*.

[91] Pons-Estel G. J., Salerni G. E., Serrano R. M. (2011). Therapeutic plasma exchange for the management of refractory systemic autoimmune diseases: report of 31 cases and review of the literature. *Autoimmunity Reviews*.

[92] Berman H., Rodríguez-Pintó I., Cervera R. (2013). Rituximab use in the catastrophic antiphospholipid syndrome: descriptive analysis of the CAPS registry patients receiving rituximab. *Autoimmunity Reviews*.

[93] Ikeda K., Nagasawa K., Horiuchi T., Tsuru T., Nishizaka H., Niho Y. (1997). C5a induces tissue factor activity on endothelial cells. *Thrombosis and Haemostasis*.

[94] Ritis K., Doumas M., Mastellos D. (2006). A novel C5a receptor-tissue factor cross-talk in neutrophils links innate immunity to coagulation pathways. *Journal of Immunology*.

[95] Fischetti F., Durigutto P., Pellis V. (2005). Thrombus formation induced by antibodies to *β*2-glycoprotein I is complement dependent and requires a priming factor. *Blood*.

[96] Schmidtko J., Peine S., El-Housseini Y., Pascual M., Meier P. (2013). Treatment of atypical hemolytic uremic syndrome and thrombotic microangiopathies: a focus on eculizumab. *American Journal of Kidney Diseases*.

[97] Lonze B. E., Singer A. L., Montgomery R. A. (2010). Eculizumab and renal transplantation in a patient with CAPS. *The New England Journal of Medicine*.

[98] Shapira I., Andrade D., Allen S. L., Salmon J. E. (2012). Brief report: induction of sustained remission in recurrent catastrophic antiphospholipid syndrome via inhibition of terminal complement with eculizumab. *Arthritis & Rheumatism*.

[99] Canaud G., Kamar N., Anglicheau D. (2013). Eculizumab improves posttransplant thrombotic microangiopathy due to antiphospholipid syndrome recurrence but fails to prevent chronic vascular changes. *American Journal of Transplantation*.

[100] Lonze B. E., Zachary A. A., Magro C. M. (2014). Eculizumab prevents recurrent antiphospholipid antibody syndrome and enables successful renal transplantation. *American Journal of Transplantation*.

[101] Morabito F., Gentile M., Gay F. (2009). Insights into defibrotide: an updated review. *Expert Opinion on Biological Therapy*.

[102] Falanga A., Vignoli A., Marchetti M., Barbui T. (2003). Defibrotide reduces procoagulant activity and increases fibrinolytic properties of endothelial cells. *Leukemia*.

[103] Asherson R. A., Cervera R., Piette J.-C. (2001). Catastrophic antiphospholipid syndrome: clues to the pathogenesis from a series of 80 patients. *Medicine*.

[104] Burcoglu-O'Ral A., Erkan D., Asherson R. (2002). Treatment of catastrophic antiphospholipid syndrome with defibrotide, a proposed vascular endothelial cell modulator. *The Journal of Rheumatology*.

[105] Gerosa M., Chighizola C., Meroni P. L. (2008). Aspirin in asymptomatic patients with confirmed positivity of antiphospholipid antibodies? Yes (in some cases). *Internal and Emergency Medicine*.

[106] Arnaud L., Mathian A., Ruffatti A. (2014). Efficacy of aspirin for the primary prevention of thrombosis in patients with antiphospholipid antibodies: an international and collaborative meta-analysis. *Autoimmunity Reviews*.

[107] Chighizola C., Schioppo T., Ingegnoli F., Meroni P. L. (2012). Potential effect of anti-inflammatory treatment on reducing the cardiovascular risk in rheumatoid arthritis. *Current Vascular Pharmacology*.

[108] Martínez F., Forner M. J., Ruano M., Abdilla N., Oltra R., García-Fuster M. J. (2006). Factors related to the risk of thrombosis in patients with lupus and antiphospholipid antibodies. *Medicina Clinica*.

[109] Shah N. M., Khamashta M. A., Atsumi T., Hughes G. R. V. (1998). Outcome of patients with anticardiolipin antibodies: a 10 year follow-up of 52 patients. *Lupus*.

[110] Wahl D. G., Bounameaux H., de Moerloose P., Sarasin F. P. (2000). Prophylactic antithrombotic therapy for patients with systemic lupus erythematosus with or without antiphospholipid antibodies: do the benefits outweigh the risks? A decision analysis. *Archives of Internal Medicine*.

[111] Wallace D. J. (1987). Does hydroxychloroquine sulfate prevent clot formation in systemic lupus erythematosus?. *Arthritis and Rheumatism*.

[112] Ruiz-Irastorza G., Egurbide M.-V., Pijoan J.-I. (2006). Effect of antimalarials on thrombosis and survival in patients with systemic lupus erythematosus. *Lupus*.

[113] Law G., Magder L., Fang H., Petri M. (2014). Hydroxychloroquine reduces thrombosis (both arterial and venous) in systemic lupus erythematosus, particularly in antiphospholipid positive patients. *Annals of the Rheumatic Diseases*.

[114] Ruiz-Irastorza G., Ramos-Casals M., Brito-Zeron P., Khamashta M. A. (2010). Clinical efficacy and side effects of antimalarials in systemic lupus erythematosus: a systematic review. *Annals of the Rheumatic Diseases*.

[115] Ostertag M. V., Liu X., Henderson V., Pierangeli S. S. (2006). A peptide that mimics the Vth region of beta-2-glycoprotein I reverses antiphospholipid-mediated thrombosis in mice. *Lupus*.

[116] de la Torre Y. M., Pregnolato F., D'Amelio F. (2012). Anti-phospholipid induced murine fetal loss: novel protective effect of a peptide targeting the *β*2 glycoprotein I phospholipid-binding site. Implications for human fetal loss. *Journal of Autoimmunity*.

[117] Ioannou Y., Romay-Penabad Z., Pericleous C. (2009). In vivo inhibition of antiphospholipid antibody-induced pathogenicity utilizing the antigenic target peptide domain I of *β*2-glycoprotein I: proof of concept. *Journal of Thrombosis and Haemostasis*.

[118] Agostinis C., Durigutto P., Sblattero D. (2014). A non-complement-fixing antibody to *β*2 glycoprotein i as a novel therapy for antiphospholipid syndrome. *Blood*.

[119] Giannakopoulos B., Krilis S. A. (2013). The pathogenesis of the antiphospholipid syndrome. *The New England Journal of Medicine*.

[120] Kolyada A., Lee C.-J., de Biasio A., Beglova N. (2010). A novel dimeric inhibitor targeting Beta2GPI in Beta2GPI/antibody complexes implicated in antiphospholipid syndrome. *PLoS ONE*.

[121] Kolyada A., Porter A., Beglova N. (2014). Inhibition of thrombotic properties of persistent autoimmune anti-*β*2GPI antibodies in the mouse model of antiphospholipid syndrome. *Blood*.

[122] Comarmond C., Cacoub P. (2013). Antiphospholipid syndrome: from pathogenesis to novel immunomodulatory therapies. *Autoimmunity Reviews*.

[123] Nishimura M., Nii T., Trimova G. (2013). The NF-*κ*B specific inhibitor DHMEQ prevents thrombus formation in a mouse model of antiphospholipid syndrome. *Journal of Nephropathology*.

[124] Pierangeli S. S., Erkan D. (2010). Antiphospholipid syndrome treatment beyond anticoagulation: are we there yet?. *Lupus*.

[125] Zhou H., Wolberg A. S., Roubey R. A. S. (2004). Characterization of monocyte tissue factor activity induced by IgG antiphospholipid antibodies and inhibition by dilazep. *Blood*.

[126] Zhou H. (2004). Dilazep and dipyridamole inhibit tissue factor expression on monocytes induced by IgG from patients with antiphospholipid syndrome. *Acta Pharmacologica Sinica*.

[127] Scoble T., Wijetilleka S., Khamashta M. A. (2011). Management of refractory anti-phospholipid syndrome. *Autoimmunity Reviews*.

[128] Jasuja R., Passam F. H., Kennedy D. R. (2012). Protein disulfide isomerase inhibitors constitute a new class of antithrombotic agents. *Journal of Clinical Investigation*.

[129] Perez-Sanchez C., Ruiz-Limon P., Aguirre M. A. (2012). Mitochondrial dysfunction in antiphospholipid syndrome: implications in the pathogenesis of the disease and effects of coenzyme Q_10_ treatment. *Blood*.

